# “Robotic-assisted surgical management of a post-brachytherapy rectoprostatic fistula: a case report”

**DOI:** 10.1186/s12894-025-01972-8

**Published:** 2025-11-10

**Authors:** Artem Goncharov, Vadim Shindyapin, Alexander Popov, Aramais Aslanyan, Emil Abdryakhimov

**Affiliations:** 1 Department of Coloproctology, Federal State Budgetary Institution “Central Clinical Hospital with Polyclinicˮ of the Administrative Directorate of the President of the Russian Federation, 15 Marshal Timoshenko Street, Moscow, 121359 Russian Federation; 2 Department of Oncourology, Federal State Budgetary Institution “Central Clinical Hospital with Polyclinicˮ of the Administrative Directorate of the President of the Russian Federation, 15 Marshal Timoshenko Street, Moscow, 121359 Russian Federation; 3 Department of Immunobiology and Biomedicine, Scientific Center of Genetics and Life Sciences, Sirius University of Science and Technology, Krasnodar Krai, Federal Territory Sirius 354340 Russia

**Keywords:** Rectoprostatic fistula, Robotic surgery, Brachytherapy complication, Case report, Coloanal anastomosis

## Abstract

**Background:**

Rectourethral fistula represents a rare but devastating complication following prostate cancer treatment, with radiation-induced fistulas presenting particular challenges due to compromised tissue vascularity and healing capacity. While various surgical approaches have been described, management of complex post-brachytherapy rectoprostatic fistulas remains challenging with limited reports of successful robotic-assisted techniques. This case report presents a novel robotic-assisted surgical approach incorporating technical modifications including rectal rotation with mesorectal interposition, which has not been widely documented in the literature for managing radiation-induced rectoprostatic fistulas.

**Case presentation:**

A 54-year-old male with localized prostate cancer (clinical stage T2N0M0, Gleason score 3 + 3) developed fecaluria, pneumaturia, and severe pelvic pain one year after brachytherapy treatment. Diagnostic imaging revealed a 1.6 cm anterior rectal wall defect with a fluid-filled cavity between the rectum and prostate. Following initial palliative sigmoidostomy for symptom relief, definitive repair was performed 13 months later using robotic-assisted radical prostatectomy with segmental rectal resection and coloanal anastomosis. Key technical innovations included bladder stabilization with externalized sutures and 180° rectal rotation, positioning the posterior mesorectum as a natural barrier against fistula recurrence. The procedure duration was 365 min with 200 mL blood loss and no immediate complications. At 24-month follow-up, the patient demonstrated complete fistula resolution with normal bowel function (Wexner continence score: 3) following stoma reversal. Prostate-specific antigen remained undetectable with no evidence of cancer recurrence. The patient reported significant quality-of-life improvement, resuming pre-illness physical activities including walking 7–10 km daily.

**Conclusions:**

This case demonstrates the feasibility and effectiveness of robotic-assisted surgical management for complex radiation-induced rectoprostatic fistulas. The novel technique of rectal rotation with mesorectal interposition may provide additional protection against recurrent fistula formation compared to conventional approaches. This innovative robotic approach could be considered in selected patients with extensive radiation-induced tissue damage where traditional repair techniques may be insufficient, potentially offering improved outcomes in this challenging clinical scenario.

**Supplementary Information:**

The online version contains supplementary material available at 10.1186/s12894-025-01972-8.

## Background

Rectourethral fistula (RUF) represents a rare but devastating complication following treatments for prostate cancer, including radiation therapy, radical prostatectomy, and brachytherapy [1, 2]. These abnormal communications between the rectum and the urinary tract significantly impact patients’ quality of life and pose considerable challenges for surgical management [1, 3]. While the overall incidence of RUF following prostate cancer treatment is relatively low, ranging from 0.1% to 3.0% according to several studies [2], the reported rate can vary by procedure with development in 0.6% to 9% of patients after radical prostatectomy [1] and approximately 1.2% after primary whole-gland cryotherapy [4]. The consequences are severe, often requiring complex reconstructive procedures with various surgical approaches (transanal, transperineal, or transabdominal) [5] and potentially resulting in permanent urinary and fecal diversions in cases with adverse features [3]. Modern management strategies increasingly involve multidisciplinary approaches and specialized techniques, including newer robotic-assisted surgical repairs that can eliminate the need for incisions through the rectal sphincter [6]. This case report describes a novel robotic-assisted surgical approach for managing a post-brachytherapy rectoprostatic fistula, a complex clinical scenario that demands multidisciplinary expertise and advanced surgical techniques.

Prostate brachytherapy remains a widely utilized curative modality for localized prostate cancer, favored for its reduced toxicity compared to radical surgery or external beam radiation therapy (EBRT) [7, 8]. Nevertheless, severe late complications, including RUF, though uncommon (incidence: 0.32%), represent clinically significant adverse events [9, 10]. RUF risk escalates with combination therapies, particularly when brachytherapy is combined with EBRT or administered post-prostatectomy [2, 11]. The underlying pathophysiology involves radiation-induced microvascular injury and delayed fibrotic transformation of peri-prostatic tissues, mechanisms that may remain subclinical for years before fistula manifestation [2, 10, 11].

Patients with rectourethral fistulas typically present with pathognomonic symptoms including fecaluria, pneumaturia, or recurrent urinary tract infections [2]. Diagnosis often requires a comprehensive approach incorporating detailed history and various imaging modalities [2, 12]. Cross-sectional imaging with retrograde cystogram or pelvic magnetic resonance imaging (MRI) is usually sufficient to delineate the fistula anatomy and identify any associated collections, but can be aided by direct visualization via cystoscopy and sigmoidoscopy for additional valuable information. Pelvic MRI can also provide an abundance of information for preoperative planning. Early diagnosis and appropriate management are important for optimal patient outcomes and to minimize potential complications [13].

The management of rectourethral fistulas remains challenging, with no universally accepted standardized approach [3, 14, 15]. Most treatment algorithms begin with fecal and/or urinary diversion, as demonstrated by multiple studies showing that temporary diverting colostomy and suprapubic cystostomy are common initial approaches [16]. Unfortunately, the majority of post-radiation fistulas require definitive surgical intervention due to compromised tissue vascularity and healing capacity, with 2021 data showing significantly lower success rates in patients with prior radiation therapy compared to those with surgery-only etiology (50% versus 100%, *p* = 0.008) [3]. Multiple surgical approaches have been described in the literature, including transanal, trans-sphincteric, transperineal, and transabdominal techniques, each with varying success rates [14, 15, 17]. According to recent 2025 data, when using gracilis muscle flap interposition, the overall success rate is approximately 68%, with notably better outcomes in patients without prior radiation therapy [18].

The complexity of rectourethral fistula repair often necessitates interposition of well-vascularized tissue between the separated rectum and urethra to minimize recurrence [5, 19]. Various flap options have been described, including gracilis muscle flaps [11, 18, 19], dartos muscle flaps [20, 21], and transanal rectal flap advancement with fibrin glue injection [22]. Gracilis muscle transposition has demonstrated good outcomes, with a recent 2025 study reporting successful fistula closure in 68% of patients following the procedure, with significantly different success rates based on prior external beam radiation therapy status [18]. Minimal complications such as main wound site infections, seroma at the harvested site of gracilis muscle flap, and urethral stricture have been reported [11]. The dartos muscle flap, harvested on a pedicle, has also been described as an option for interposition [20, 21]. A multidisciplinary approach involving urology, colorectal, and plastic surgery teams has been recommended for optimal management of these complex cases [5, 19].

In recent years, minimally invasive approaches, particularly robotic-assisted techniques, have emerged as promising alternatives for rectourethral fistula repair [23, 24]. The robotic platform offers several advantages, including superior visualization, enhanced dexterity, and improved ergonomics, especially in the confined spaces of the deep pelvis [12, 25]. Various robotic approaches have been described, including transabdominal and transanal techniques [6]. The robotic transanal minimally invasive surgery (TAMIS) approach represents a novel technique that potentially combines the benefits of minimally invasive surgery with excellent visualization of the fistula site [24, 26]. This approach is particularly suitable for selected cases, avoiding external incisions through the rectal sphincter [6] and potentially enhancing postoperative recovery time [27].

The prognosis following rectourethral fistula repair largely depends on several factors, including fistula etiology, size, prior radiation exposure, and surgical technique. Radiation-induced fistulas present particular challenges, with prior radiotherapy significantly associated with more complex and persistent fistulas [12]. Multiple studies have highlighted the impact of prior radiotherapy and ablative therapy on surgical outcomes for rectourethral fistulas, emphasizing the need for specialized approaches in these complex cases [5, 28]. Research indicates that larger fistulas (>2 cm) and those with extensive surrounding tissue damage tend to have poorer outcomes and higher recurrence rates, with fistulas larger than 3 cm often considered nonrepairable through standard techniques and requiring more extensive surgical interventions such as pelvic exenteration [5]. Contemporary algorithmic approaches to rectourethral fistula management demonstrate improved outcomes when treatment is tailored to specific patient factors, with newer robotic surgical techniques showing promise for certain cases.

The case presented in this report is unique in several aspects. First, it involves a post-brachytherapy rectoprostatic fistula, a relatively uncommon complication that presents significant management challenges due to radiation-induced tissue changes. Second, the surgical approach employed combines robotic-assisted prostatectomy with segmental rectal resection and coloanal anastomosis, representing an innovative technique not widely reported in the literature. Third, several technical modifications were implemented to address the specific challenges encountered, including the 180° rotation of the rectum beneath the cystourethral anastomosis to allow the posterior mesorectum to tamponade the anastomotic site, potentially reducing the risk of urinary fistula recurrence.

The complex nature of post-radiation rectourethral fistulas often necessitates individualized approaches tailored to the specific patient characteristics and fistula anatomy [5, 19, 29]. While conservative management with fecal and urinary diversion may be successful in selected, uncomplicated cases, most radiation-induced fistulas require definitive surgical repair, with reported success rates of approximately 68% for gracilis flap reconstruction in radiated patients [18, 30]. The optimal timing of surgical intervention typically involves initial diversion followed by definitive repair after approximately 3 months, allowing for resolution of acute inflammation and optimization of tissue quality [11]. Algorithmic, multidisciplinary approaches to rectourethral fistula management have demonstrated improved outcomes, with one study reporting a 33% increase in definitive healing rates following implementation of a structured treatment protocol [5, 19]. Recent advances include robotic surgical techniques and careful postoperative management to counteract radiation-induced tissue damage [31, 32].

The case reported herein contributes to the growing body of literature on minimally invasive approaches for complex rectourethral fistula repair. By describing the technical details, challenges encountered, and successful outcomes achieved, this report aims to enhance the surgical armamentarium available for managing this challenging complication. Furthermore, the long-term follow-up data provided offers valuable insights into the durability of repair and potential complications that may arise during the postoperative period.

In conclusion, rectourethral fistula following brachytherapy for prostate cancer represents a rare but serious complication that requires a multidisciplinary approach and often complex surgical reconstruction. The evolving landscape of minimally invasive surgery, particularly robotic-assisted techniques, offers promising alternatives for managing these challenging cases. This case report details an innovative robotic-assisted approach for the successful management of a post-brachytherapy rectoprostatic fistula, providing valuable insights for surgeons facing similar clinical scenarios.

## Case presentation

A 54-year-old male presented to our institution with a complex medical history following treatment for prostate cancer. Diagnosed in 2017 with localized prostate cancer (stage cT2N0M0, Gleason score 3 + 3), he underwent brachytherapy at the Federal State Budgetary Healthcare Institution “Clinical Hospital No. 8” in Obninsk, Russia. Prior to brachytherapy, the prostate biopsy was performed via a transperineal approach. Additionally, no rectal spacer (such as SpaceOAR hydrogel) was used during the brachytherapy procedure. He had no significant comorbidities, a body mass index of 32 indicating obesity class I, and a background as a physically active individual with a history of competitive judo (master of sports) and a managerial occupation involving moderate physical labor. He had no significant family history of hereditary or chronic conditions. Approximately one year post-brachytherapy, in 2018, he developed distressing symptoms including fecaluria, pneumaturia, and severe pelvic pain exacerbated during defecation, which he described as “unbearable, like passing shattered glass”. These symptoms progressively worsened, with continuous urinary leakage via the rectum over six months, significantly impacting his quality of life and requiring opioid analgesics for pain management.

The diagnostic journey was challenging due to initial misattribution of symptoms to post-brachytherapy proctitis, delaying the identification of a rectourethral fistula by nearly 10 months. A pelvic MRI in 2018 revealed a 1.6 cm defect in the anterior rectal wall with a fluid-filled cavity (3.3 × 2.5 × 1.9 cm) between the rectum and prostate, alongside loss of tissue planes at the rectourethral interface. Cystoscopy further confirmed a fistulous opening in the prostatic urethra surrounded by edematous mucosa, solidifying the diagnosis of a rectourethral fistula as a complication of radiation therapy. The complexity of the anatomy, compounded by radiation-induced fibrosis, posed additional difficulties in imaging interpretation and planning subsequent interventions.

In response to the severity of symptoms and to mitigate further complications, a laparoscopic sigmoidostomy was performed in November 2018, with the stoma placed in the lumbar region along the posterior axillary line. This intervention markedly alleviated the patient’s pain and improved his emotional and physical state, allowing a return to moderate physical activity despite the inconvenience of the stoma, particularly during summer months. However, the persistent desire to restore normal bowel continuity and return to his pre-illness quality of life prompted further referral for definitive management.

In 2019, the patient was referred to the Department of Oncourology at the Federal State Budgetary Institution “Central Clinical Hospital with Polyclinic” of the Administrative Department of the President of the Russian Federation for specialized care. After thorough multidisciplinary evaluation, a robotic-assisted radical prostatectomy and segmental rectal resection with coloanal anastomosis were performed in December 2019, preserving the existing sigmoidostomy. Preoperative management included standard antibiotic prophylaxis, thromboprophylaxis, and mechanical bowel preparation. The procedure was conducted under endotracheal anesthesia, with the patient positioned supine in a lithotomy position using a split-leg table. The surgical field was prepped and draped, with a colostomy bag secured to the pre-existing double-barrel sigmostomy.

**Fig. 1:**
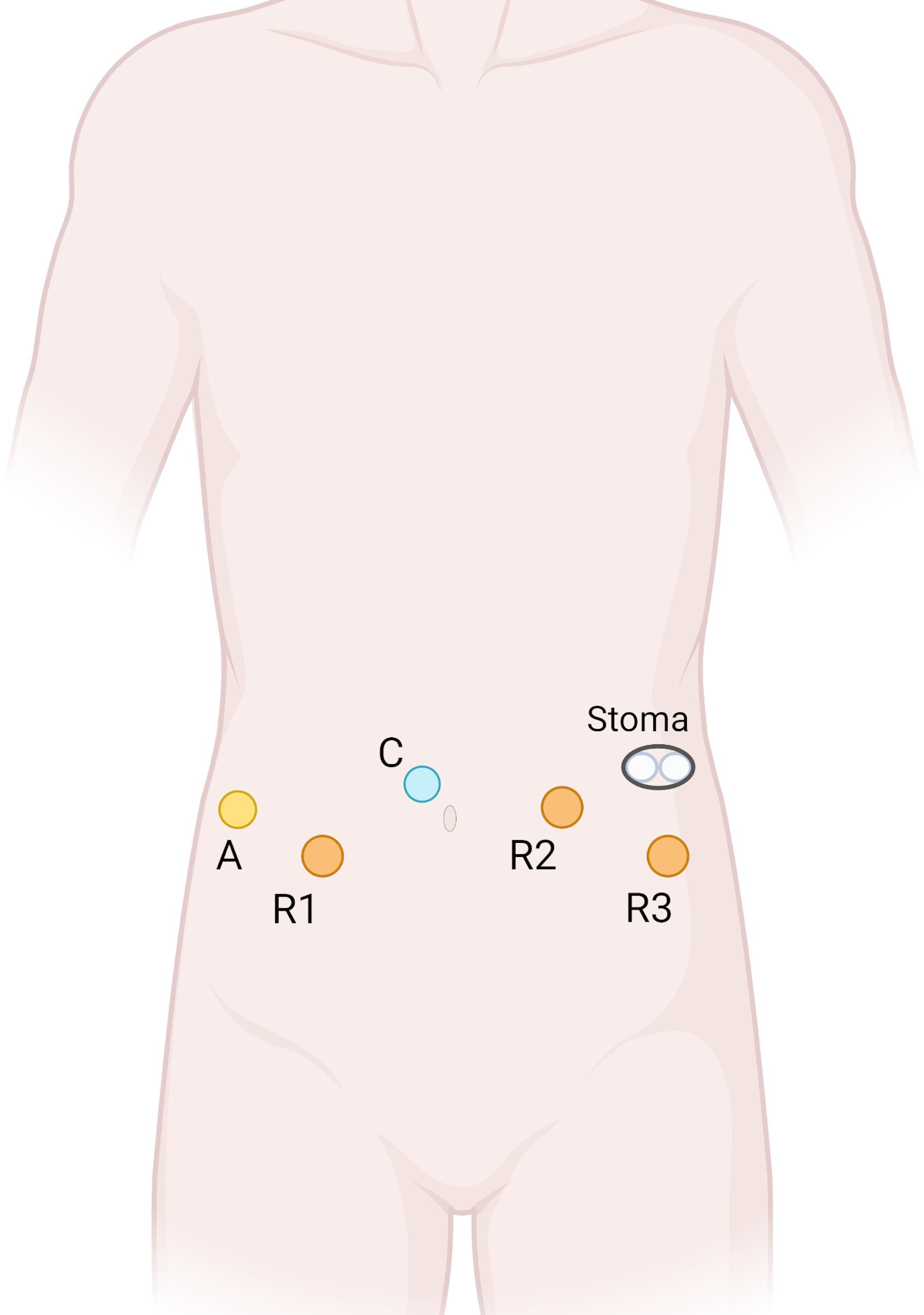
Trocar placement. A- assistant port; С - camera port; R1, R2, R3 - robotic ports

The rationale for choosing a robotic approach centered on enhanced visualization and precision in the deep, irradiated pelvis, crucial for managing the extensive defect and minimizing recurrence risk. Trocar placement (illustrated in Fig. 1) was standardized for both urological and colorectal phases, utilizing the da Vinci Si robotic system.

**Fig. 2:**
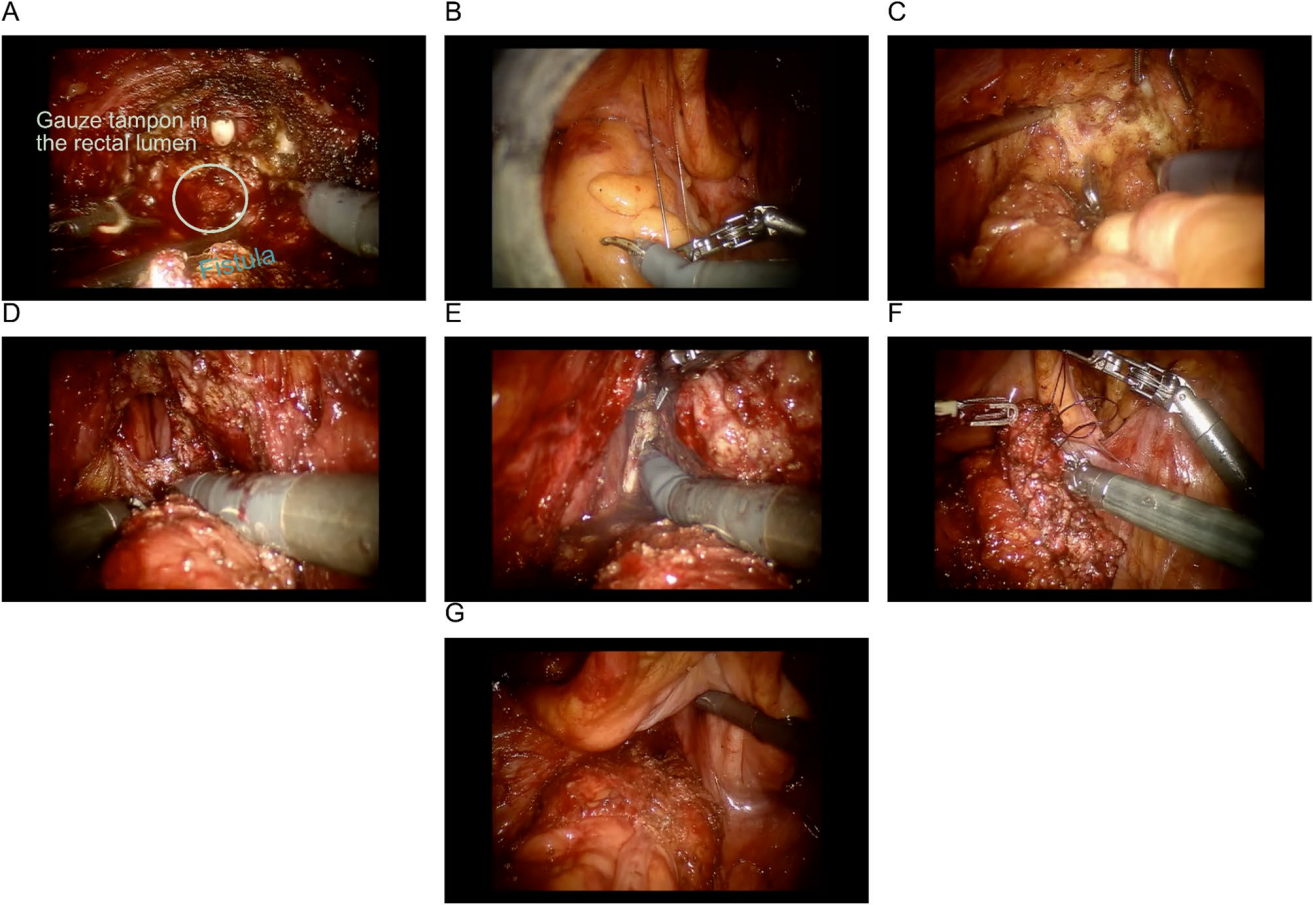
Operational images. **A** - prostate removed; rectal fistula visualized with a tampon/packing in the bowel lumen; **B** - temporary fixation of the mobile bladder with a stay suture to improve rectal exposure; **C** - anterior rectal wall dissection; **D** - exposure of the rectal fistula; **E** - transabdominal division of the rectum distal to the fistula; **F** - closure of the distal rectal stump in preparation for transanal pull-through; **G** - transanal pull-through of the mobilized rectum for segmental resection and creation of a coloanal anastomosis with 180° rectal rotation

Surgical Technique (operational images are shown in the Fig. 2):

1. Urological Phase:
  The urological team initiated dissection in the prevesical space. Following bladder mobilization, access to the anterior prostate surface was achieved. Given the absence of clear prostatic boundaries, the endopelvic fascia was incised, revealing the fistulous tract. Dissection between the bladder neck, prostate, and membranous urethra allowed complete prostate removal, exposing the fistulous tract.
2. Colorectal Transabdominal Phase:
  Using monopolar scissors, medial peritoneal incision along the sigmoid mesentery enabled mediolateral mobilization of the sigmoid and rectum while preserving the inferior mesenteric vessels. Rectal mobilization extended to the pelvic floor, with notable perifistular scar tissue observed during anterior dissection. The rectum was sharply transected circumferentially distal to the fistulous defect.
3. Colorectal Perineal Phase:
  The da Vinci console was temporarily disconnected, and the patient was repositioned into lithotomy. A Lone Star retractor facilitated exposure of the low rectum. The mobilized rectum and sigmoid colon were exteriorized transanally, followed by circular segmental resection of the affected rectal segment for histopathological analysis. Prior to coloanal anastomosis, a 180° rotation of the mesorectum positioned its fascia as a natural barrier between the prostatic bed and anastomoses to prevent fistulation. The coloanal anastomosis was completed with a single-layer interrupted 3 − 0 Vicryl suture. A Jackson-Pratt 8Fr drain was placed transperineally in the presacral space.
4. Urethrovesical Anastomosis Phase:
  Following reconnection of the da Vinci console, the urethrovesical anastomosis was constructed using two separate 3-0 Vloc sutures. An indwelling urinary catheter was placed, and anastomotic integrity was confirmed intraoperatively. The urinary catheter remained in situ for 30 postoperative days to ensure healing.

Intraoperative challenges included severe inflammatory changes and a significant rectal wall defect with an associated cavity, necessitating a resectional approach over local reconstruction. Key technical modifications included stabilizing the mobilized bladder with externalized sutures, mesorectal rotation for fistula prophylaxis, and employing the Natural Orifice Specimen Extraction (NOSE) technique to avoid additional peritoneal incisions.

The surgery lasted 365 min with a blood loss of 200 mL. The patient was discharged on postoperative day 8 without immediate complications.

**Fig. 3:**
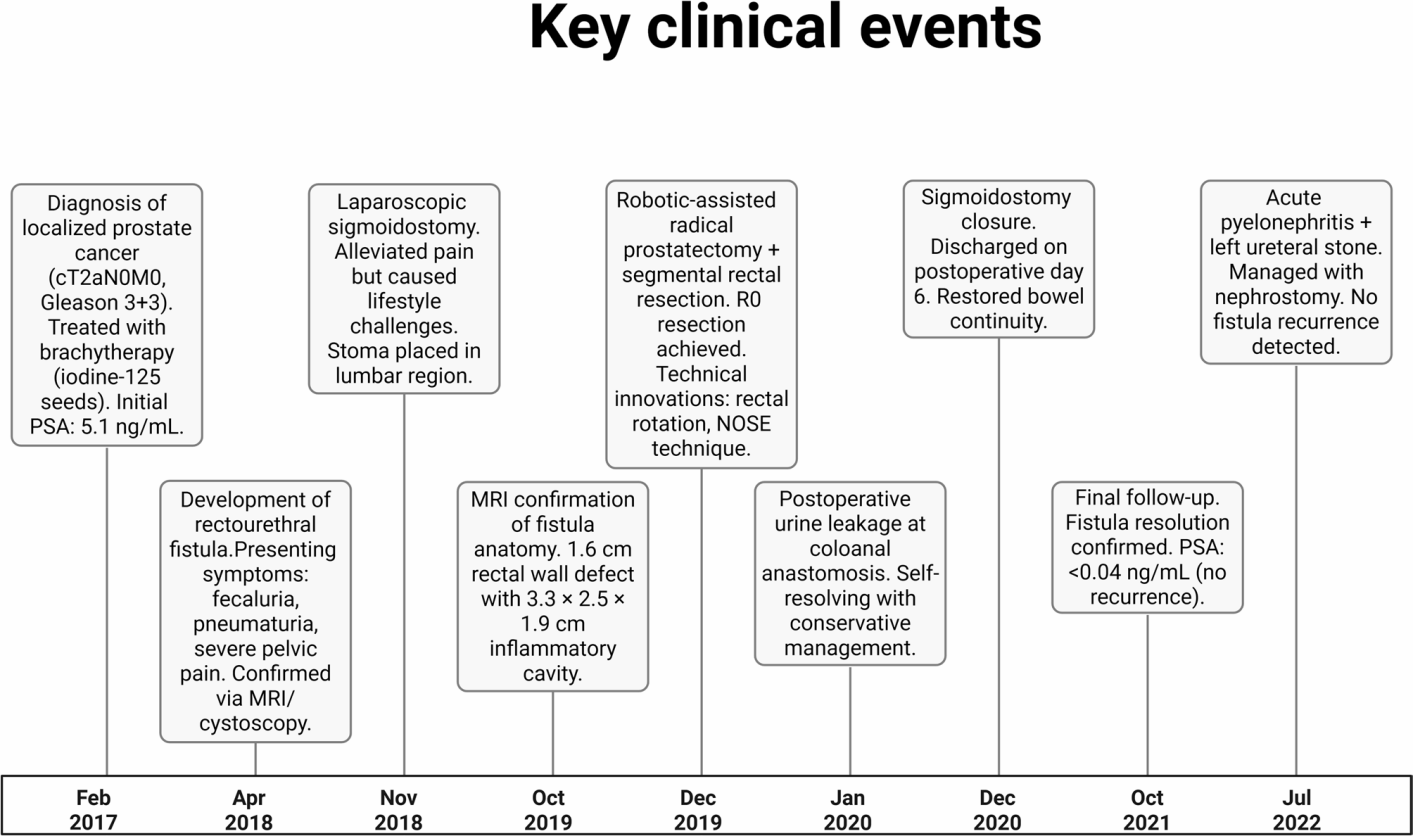
Key clinical events (2017–2022). Brachytherapy complications: fistula development linked to radiation-induced fibrosis. Surgical innovations: rectal rotation technique reduced urinary leakage risk by utilizing mesorectum as a natural barrier. Long-term outcomes: 24-month follow-up demonstrated restored urinary/bowel function and cancer-free status

**Fig. 4:**
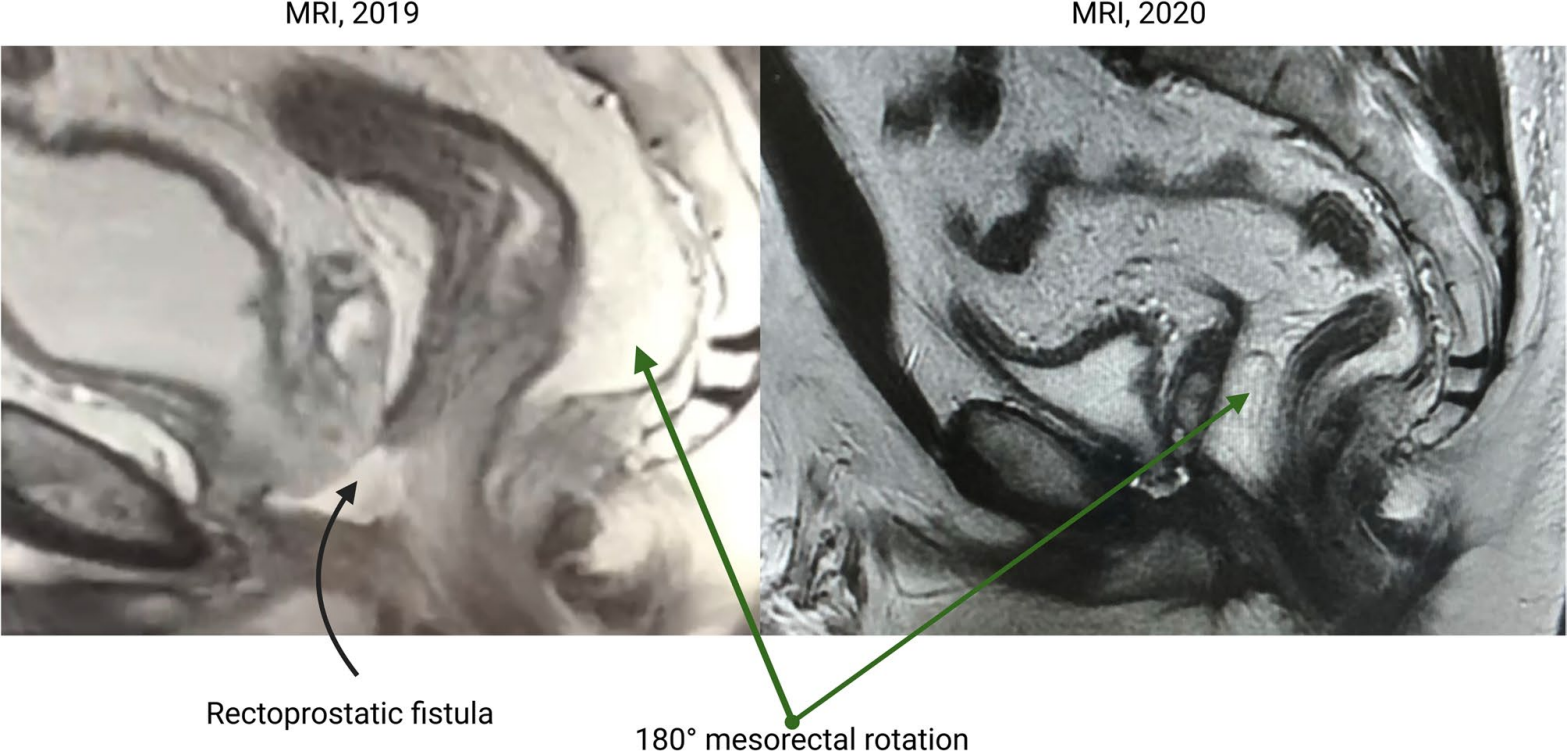
Comparative MRI scans from 2019 and 2020. The 2019 scan reveals a rectoprostatic fistula, while the 2020 scan demonstrates a 180° mesorectal rotation

The postoperative course was closely monitored (Fig. 3). An MRI in 2020 showed the rectum positioned with the mesorectum adjacent to the cystourethral anastomosis, with minor urine extravasations unrelated to the coloanal anastomosis noted (Fig. 4). A self-resolving urine leakage at the coloanal anastomosis was documented in January 2020. By December 2020, the sigmoidostomy was successfully closed, and the patient was discharged on postoperative day 6 without complications. Follow-up in October 2021 confirmed complete resolution of the fistula and absence of urinary leakage, with restored bowel continuity and normal sphincter function (Wexner score: 3). PSA levels remained undetectable (< 0.04 ng/mL), indicating no evidence of cancer recurrence.

### Patient perspective, treatment experience and recovery journey

The patient described the initial development of rectoprostatic fistula following brachytherapy as “a life-altering ordeal,” marked by debilitating urinary leakage through the rectum and excruciating pain during bowel movements. “The constant pain felt unbearable-like a burning sensation that no medication could fully control,” he recalled, emphasizing reliance on high-dose analgesics for temporary relief. The decision to undergo sigmoidostomy in 2018 brought immediate relief: “The stoma eliminated the agony. For the first time in months, I could function without dread.” Post-stoma, he reported significant improvements in emotional well-being and physical activity, resuming daily walks (7–10 km/day) and light occupational tasks.

Despite these gains, stoma maintenance proved challenging, particularly during summer months: “The inconvenience and self-consciousness made me desperate for definitive repair.” The robotic-assisted surgery in 2019 was described as “a leap of faith,” with the patient crediting the surgical team’s expertise for restoring normal bowel function post-stoma closure. He highlighted the importance of gradual physical rehabilitation, noting, “I cautiously exceeded medical restrictions to rebuild strength-returning to judo training was my motivation.”

### Emotional and psychological impact

The psychological toll of recurrent symptoms prior to stoma placement emerged as a central theme: “The fear of permanent disability nearly broke me. Visiting Mount Athos became a spiritual anchor during that darkness.” Post-recovery, he reflected, “Hope was my lifeline - believing in the team’s skill kept me fighting.”

### Treatment reflections and outcomes

While expressing profound gratitude toward the surgical team, he voiced retrospective doubts about brachytherapy: “Had I known the fistula risk, I might have chosen prostatectomy initially.” At 24-month follow-up, he reported complete resolution of symptoms and a return to pre-illness activity levels, concluding, “Today, I feel reborn - no reminders of that nightmare remain.”

## Discussion

RUF following prostate cancer treatment represents a devastating complication with profound implications for patient quality of life [33]. This case demonstrates a successful robotic repair of a complex radiation-induced fistula using novel techniques. The patient’s restored urinary and bowel normal function highlights this as a viable minimally invasive option for this serious complication.

### Epidemiology and etiology

The timing of fistula development in our patient (approximately one year post-brachytherapy) is consistent with published literature, which documents cases developing up to 23 months after seed implantation [34]. This delayed presentation underscores the importance of long-term surveillance following radiation therapy and highlights the need for prompt recognition of suspicious symptoms such as fecaluria and pneumaturia [2].

The pathophysiology of radiation-induced fistula formation involves progressive vascular damage, tissue hypoxia, and subsequent fibrosis that may manifest months to years following treatment [35, 36]. Radiation therapy causes damage to the epithelium through secondary ionizing radiation, leading to compromised healing capacity in irradiated tissues [37]. This makes radiation-induced fistulas particularly challenging to repair.

The fistula’s development, despite no technical error (i.e. the type of biopsy performed, needle misplacement; not using a rectal spacer, such as SpaceOAR hydrogel, during the brachytherapy procedure; recent case reports have documented rectourethral fistula formation as a rare but serious complication following SpaceOAR placement, particularly when the hydrogel is inadvertently injected into the rectal wall [32, 38]), could be attributed to a combination of patient-specific anatomical factors and the inherent challenges of brachytherapy dosimetry. The patient’s class I obesity may have altered the pelvic anatomy [39], potentially bringing the anterior rectal wall closer to the prostate gland than anticipated and increasing its susceptibility to radiation exposure. Even with precise seed placement, dose inhomogeneities (“hot spots”) at the prostate-rectal interface can cause focal tissue necrosis and subsequent fistula formation - a risk difficult to completely eliminate.

### Diagnostic challenges

The diagnosis of post-radiation RUF presents unique challenges, as evidenced in our case where initial symptoms were misattributed to radiation proctitis, delaying proper diagnosis by nearly 10 months. This delay is not uncommon in the literature, with several studies noting similar diagnostic challenges in radiation-induced fistulas [40–42]. One study reported diagnostic delays of more than 3 months in five patients with urosymphyseal fistulas after pelvic radiotherapy [40], while another documented delayed rectourethral fistula development occurring 9 months after completion of radiotherapy [42]. The progressive nature of radiation-induced tissue damage often results in subtle initial symptoms that may be mistaken for more common complications such as radiation proctitis or cystitis [41, 43]. Chronic radiation induces widespread intestinal collagen deposition and fibrosis along with occlusive vasculitis in small arterioles, effects that may develop over several months or years, resulting in bowel strictures, ulcerations, and fistulas [41]. Our diagnostic approach ultimately involved a combination of MRI imaging and cystoscopy, which aligns with current best practices [2, 12]. The MRI findings in our case - specifically the 1.6 cm defect in the anterior rectal wall with an associated inflammatory cavity - were instrumental in guiding our surgical approach.

### Surgical management considerations

The management of rectourethral fistulas remains one of the most challenging scenarios in reconstructive urology [14, 19, 44]. While conservative management with urinary and fecal diversion may be sufficient for small, simple fistulas [30], radiation-induced fistulas almost invariably require definitive surgical intervention due to the compromised tissue vascularity [44, 45]. In our case, initial management with sigmoidostomy provided symptomatic relief but, as expected, did not result in spontaneous fistula closure, necessitating definitive repair.

Multiple surgical approaches have been described for RUF repair, including transanal, trans-sphincteric, transabdominal, and transperineal techniques [14]. The York-Mason posterior trans-sphincteric approach has traditionally been favored due to its excellent exposure of the fistula site [9], though concerns regarding potential sphincter dysfunction have prompted exploration of alternative approaches. The transperineal approach has gained popularity, with studies reporting high success rates for acquired rectourethral fistulas [14].

In recent years, minimally invasive approaches, particularly robotic-assisted techniques, have emerged as promising alternatives for RUF repair. The robotic platform offers several advantages for deep pelvic surgery, including enhanced visualization, improved dexterity, and superior ergonomics. Alternative robotic approaches include transabdominal techniques with omental flap interposition, as described by Brassetti et al., who reported successful outcomes using wide cystotomy and omental barrier placement between rectum and bladder [46]. However, our technique of mesorectal rotation may offer advantages in radiation-damaged tissue where omental mobilization might be challenging. Various robotic approaches have been described, including transabdominal and the more recent TAMIS techniques [24].

### Rationale for our surgical approach

Our decision to employ an aggressive robotic-assisted radical prostatectomy and segmental rectal resection was driven by case-specific factors: the extensive radiation-induced tissue damage (1.6 cm defect), severely compromised tissue quality that precluded local repair, and the opportunity to simultaneously address both the fistula and potential oncologic risks in the irradiated field.

The technical modifications employed in our case merit particular attention. The 180° rotation of the rectum beneath the cystourethral anastomosis to position the posterior mesorectum as a natural barrier represents an innovative approach that may have contributed to the successful outcome.While gracilis muscle flap interposition remains the most widely established technique for complex rectourethral fistula repair with consistent success rates reported across multiple series, our mesorectal rotation technique offers several distinct advantages over conventional interposition methods. Unlike gracilis flaps that require additional surgical sites and specialized plastic surgery expertise [18, 19], mesorectal rotation utilizes naturally available tissue that requires no additional mobilization beyond the planned rectal resection. Furthermore, while omental flap interposition requires adequate omental length and may not be feasible in all patients [47], the mesorectum provides a consistent, well-vascularized tissue barrier positioned anatomically optimal for reinforcing the prostatic bed anastomosis. This technique has not been widely reported in the literature but follows sound surgical principles by utilizing well-vascularized tissue for reinforcement of the repair site.

### Comparison with existing literature

The outcomes achieved in this case demonstrate favorable alignment with contemporary reports on RUF repair. Recent studies confirm that prior radiotherapy significantly increases surgical complexity and recurrence rates for radiation-induced fistulas due to compromised tissue vascularity and fibrosis [18, 19]. For example, a 2025 cohort study of 22 patients undergoing gracilis flap repair reported a 68% success rate in radiated patients, underscoring the challenges of radiation-damaged tissues [18]. Despite these obstacles, the presented case achieved complete fistula resolution and restoration of urinary/bowel function, consistent with outcomes from specialized robotic approaches in similarly complex scenarios.

The robotic TAMIS approach referenced in the literature shares key technical advantages with our method, particularly enhanced visualization and precision in deep pelvic dissection [48]. However, this case diverges through 180° rectal rotation utilizing the mesorectum as a natural barrier against urinary leakage, a technique not widely documented in prior robotic RUF repairs. These modifications align with emerging principles of interposing well-vascularized tissue in radiation-damaged fields [19, 49]. While traditional approaches utilize omental flaps for tissue interposition, our mesorectal rotation technique provides a more anatomically positioned barrier that requires no additional mobilization, potentially reducing operative complexity compared to conventional omental transposition methods [46].

The impact of prior radiation therapy on surgical outcomes cannot be overstated. Beddy et al. demonstrated that radiation significantly increases the complexity of fistula repair and may necessitate more aggressive surgical approaches [50]. Our decision to perform segmental rectal resection rather than local repair aligns with this understanding of radiation-induced tissue damage.

### Advantages and limitations of our approach

The robotic approach employed in our case provided enhanced visualization in the distorted irradiated field, precision in dissection and reconstruction, and allowed simultaneous oncologic and reconstructive surgery with minimized incisions (NOSE technique). However, its limitations include high technical complexity requiring advanced expertise in robotic surgery and specialized equipment, an aggressive nature unsuitable for frail patients, limited long-term data, and extended operative time challenging in resource-limited settings.

The 365-minute duration reflects the sequential completion of two major procedures by dedicated urological and colorectal teams. Contemporary data report mean operative times of 264.4 ± 57.3 min for robotic total mesorectal excision (crossover docking) and 235.5 ± 53.9 min for standard docking, respectively, in robotic rectal resections [51], and 105 ± 20.4 min (range 80–150 min) for robot-assisted radical prostatectomy completed without conversion or major intraoperative injuries [52, 53]. Taken together, an expected aggregate duration of approximately 315–415 min for these sequential procedures is consistent with our total operative time of 365 min, supporting acceptable efficiency for this dual-specialty approach.

While this report details a single case, the technical principles employed align with the evolving paradigm in robotic pelvic reconstruction. The critical step of well-vascularized tissue interposition is a cornerstone of successful fistula repair, particularly in irradiated fields [19]. Our technique of mesorectal rotation provides a novel method to achieve this interposition without requiring additional flap harvesting, potentially reducing morbidity. Recent literature supports the feasibility and advantages of robotic approaches for complex fistulae, reporting high success rates and emphasizing the importance of precision in dissection and reconstruction [24, 48]. Although multi-institutional studies are needed to establish standardized protocols, this case contributes to the growing evidence that robotic-assisted techniques, especially those incorporating innovative interposition strategies, are a viable and promising option for managing these devastating complications.

### Long-term outcomes and prognosis

At 24-month follow-up, the patient demonstrated sustained recovery with no fistula recurrence and excellent urinary/bowel function. A Wexner score of 3 indicated minimal bowel impact despite extensive initial pathology. During the active fistula phase, standard scoring was not applicable due to simultaneous urinary drainage through the urethra and rectal fistula; the condition was qualitatively categorized as severe. Urinary function (ICIQ-SF) improved from 11 points at 3 months to 6 points at 24 months, indicating clinically meaningful recovery [54]. Sexual function showed gradual multidimensional recovery: complete inactivity initially, with returned libido by 30 months though erectile attempts remained limited by pain and dysfunction.

The patient’s perspective highlights significant functional recovery, including resuming daily walks of 7–10 km, and a profoundly improved quality of life described as a “rebirth.” Additionally, undetectable PSA levels confirmed ongoing cancer control, supporting the dual benefit of our radical surgical approach.

### Case-specific challenges and technical pearls

Several aspects of our approach deserve emphasis as potential “pearls” for surgeons facing similar challenging cases:

1. The utilization of externalizing sutures to stabilize the mobilized bladder, enhancing exposure during the deep pelvic dissection.
2. The 180° rotation of the rectum to position the mesorectum against the cystourethral anastomosis, providing a natural tissue barrier.
3. The application of the NOSE technique to avoid additional peritoneal incisions, potentially reducing postoperative adhesions and pain.
4. The self-retaining Lone Star retractor for the perineal phase, which provides consistent exposure without requiring an assistant.

These technical modifications may have broader applicability in other complex pelvic reconstructive procedures beyond rectourethral fistula repair.

### Clinical implications and future directions

Our robotic-assisted approach successfully managed a complex radiation-induced fistula, though it may not suit all cases. Future research requires prospective multicenter studies, standardized treatment algorithms, investigation of bioengineered grafts, and long-term quality-of-life assessments.

### Limitations of the case report

The findings of this single case are not universally generalizable. Limitations include the use of a specialized robotic platform, an aggressive surgical approach unsuitable for all patients, and a follow-up period that may not capture very late complications.

## Conclusion

This case demonstrates successful robotic management of a complex post-brachytherapy fistula using novel technical modifications that combined oncologic and reconstructive principles. The procedure achieved complete fistula resolution with excellent functional outcomes and restored quality of life, highlighting the potential of robotic techniques for complex pelvic reconstruction. While not universally applicable, our approach aligns with established repair principles using well-vascularized tissue interposition through minimally invasive surgery. Future research should focus on standardizing approaches, comparing techniques prospectively, and improving early detection to enable simpler interventions.

## Supplementary Information

Supplementary Material 1.

## Data Availability

The data supporting the conclusions of this article are included within the article. Additional raw data supporting the findings are available from the corresponding author upon reasonable request.

## Abbreviations

cm: Centimeters
EBRT: External beam radiation therapy
ICIQ: SF-International Consultation on Incontinence Questionnaire Short Form
MRI: Magnetic resonance imaging
NOSE: Natural orifice specimen extraction
PSA: Prostate-specific antigen
RUF: Rectourethral fistula
TAMIS: Transanal minimally invasive surgery
