# Supplementary material for: “Robotic-assisted surgical management of a post-brachytherapy rectoprostatic fistula: a case report”
Source: BMC Urol. 2025 Nov 10;25:278. doi: 10.1186/s12894-025-01972-8 (PMC12599079; doi:10.1186/s12894-025-01972-8)
Supplement: Supplementary file 1 — Supplementary Material 1. [file 12894_2025_1972_MOESM1_ESM.pdf]

CARE case report guidelines

CARE Checklist of information to include when writing a case report

(cc) BY-NC-ND

Topic	Item	Checklist item description	Reported on Line
**Title**	1	The diagnosis or intervention of primary focus followed by the words "case report"	1 - 2
**Key Words**	2	2 to 5 key words that identify diagnoses or interventions in this case report, including "case report"	16 - 17
**Abstract (no references)**	3a	Introduction: What is unique about this case and what does it add to the scientific literature?	19 - 27
	3b	Main symptoms and/or important clinical findings	28 - 31
	3c	The main diagnoses, therapeutic interventions, and outcomes	31 - 41
	3d	Conclusion—What is the main "take-away" lesson(s) from this case?	42 - 48
**Introduction**	4	One or two paragraphs summarizing why this case is unique (**may include** references)	133 - 160
**Patient Information**	5a	De-identified patient specific information	169 - 170
	5b	Primary concerns and symptoms of the patient	178 - 183
	5c	Medical, family, and psycho-social history including relevant genetic information	174 - 178
	5d	Relevant past interventions with outcomes	170 - 174
**Clinical Findings**	6	Describe significant physical examination (PE) and important clinical findings	186 - 190
**Timeline**	7	Historical and current information from this episode of care organized as a timeline	267 - 272
**Diagnostic Assessment**	8a	Diagnostic testing (such as PE, laboratory testing, imaging, surveys).	186 - 190
	8b	Diagnostic challenges (such as access to testing, financial, or cultural)	184 -186
	8c	Diagnosis (including other diagnoses considered)	188 - 192
	8d	Prognosis (such as staging in oncology) where applicable	120 - 123
**Therapeutic Intervention**	9a	Types of therapeutic intervention (such as pharmacologic, surgical, preventive, self-care)	202 - 204
	9b	Administration of therapeutic intervention (such as dosage, strength, duration)	256 - 257
	9c	Changes in therapeutic intervention (with rationale)	193 - 194, 198 - 199
**Follow-up and Outcomes**	10a	Clinician and patient-assessed outcomes (if available)	263 - 266
	10b	Important follow-up diagnostic and other test results	258 - 260
	10c	Intervention adherence and tolerability (How was this assessed?)	287 - 292, 301-303
	10d	Adverse and unanticipated events	259 - 261
**Discussion**	11a	A scientific discussion of the strengths AND limitations associated with this case report	462 - 474, 480 - 483
	11b	Discussion of the relevant medical literature **with references**	304 - 483
	11c	The scientific rationale for any conclusions (including assessment of possible causes)	374 - 379
	11d	The primary "take-away" lessons of this case report (without references) in a one paragraph conclusion	485 - 493
**Patient Perspective**	12	The patient should share their perspective in one to two paragraphs on the treatment(s) they received	276 - 303

**Informed Consent**	**13**	Did the patient give informed consent? Please provide if requested	**Yes** ✓ **No** ☐
